# Elevação Enganosa do Segmento ST Ântero-Septal e Padrão de Brugada Causado por Oclusão Isolada da Artéria do Cone

**DOI:** 10.36660/abc.20230749

**Published:** 2024-07-01

**Authors:** Hakan Gökalp Uzun, İrfan Özgen

**Affiliations:** 1 Izmir Tepecik Training and Research Hospital Department of Cardiology Konak İzmir Turquia Izmir Tepecik Training and Research Hospital – Department of Cardiology, Konak, İzmir – Turquia

**Keywords:** Obstrução da Via de Saída Ventricular Direita, Infarto do Miocárdio com Supradesnível do Segmento ST, Síndrome de Brugada

## Abstract

A artéria do cone (AC) irriga a via de saída do ventrículo direito (VSVD). A elevação do segmento ST nas derivações V1-3, que pode assemelhar-se aos padrões de eletrocardiograma (ECG) de Brugada, foi relatada devido à oclusão da AC. Um paciente do sexo masculino, 68 anos de idade, foi internado no hospital com diagnóstico de infarto do miocárdio sem supradesnivelamento do segmento ST. Uma angiografia coronária revelou uma dissecção na AC, provavelmente causada pelo cateter. Devido ao pequeno calibre da AC, a terapia medicamentosa foi escolhida como curso de ação. No entanto, após o procedimento, um ECG mostrou alterações consistentes com características dos padrões de Brugada tipo 1 e tipo 2, com elevações do segmento ST nas derivações V1-4. A imagem coronariana subsequente revelou que a AC havia progredido para oclusão total. Apesar das diversas tentativas de obter a reentrada no lúmen verdadeiro, não houve êxito. Com base na relação risco-benefício, foi tomada a decisão de continuar com a terapia medicamentosa. Este é o primeiro caso relatado de oclusão da AC induzida por dissecção por cateter, que se manifesta como elevação ântero-septal do segmento ST. O paciente não relatou sintomas anginosos ou eventos arrítmicos, o que contrasta com o conhecimento convencional. Nem todas as obstruções da AC ou infartos da VSVD causam padrões semelhantes aos de Brugada. Quando isso ocorre, as elevações de ST tendem a ser menores do que as da verdadeira síndrome de Brugada.

## Introdução

A artéria do cone (AC), frequentemente o primeiro ramo da artéria coronária direita (ACD) com prevalência de 80,5%, supre a via de saída ou cone arterial (infundíbulo) do ventrículo direito (VD), uma região considerada propensa à geração de arritmias.^1,2^

A elevação do segmento ST nas derivações V1-3, por vezes semelhante aos padrões de ECG de Brugada, foi relatada devido à interrupção do fluxo sanguíneo para a AC através de vários mecanismos.^3^

## Relato de Caso

Paciente do sexo masculino, 68 anos, ex-fumante, sem histórico de doença crônica ou uso de drogas, foi admitido na unidade coronariana (UCO) com queixas de desconforto respiratório, palpitações e inchaço nas pernas há 10 dias.

**Figura 1:**
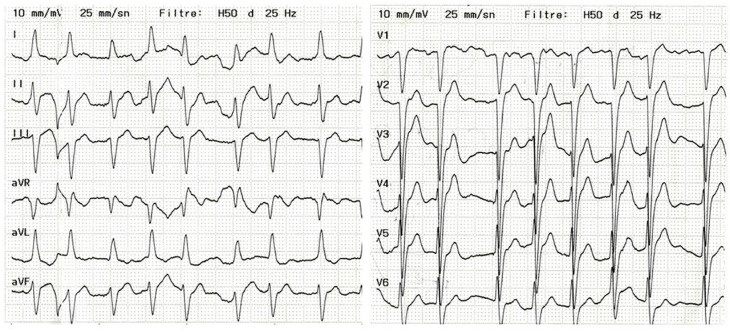
– O ECG de admissão mostrou fibrilação atrial, má progressão da onda R nas derivações precordiais, sobrecarga ventricular esquerda e bloqueio fascicular anterior esquerdo.

Os sinais vitais eram normais, exceto por uma frequência cardíaca irregular e rápida, que foi posteriormente confirmada no ECG como consistente com fibrilação atrial com resposta ventricular rápida. O ECG de admissão revelou fibrilação atrial, má progressão da onda R nas derivações precordiais, sobrecarga ventricular esquerda e bloqueio fascicular anterior esquerdo (Figura 1). Estertores confinados às bases pulmonares eram audíveis à ausculta. O ecocardiograma transtorácico, subótimo devido à taquicardia subjacente, mostrou hipocinesia global leve com fração de ejeção do ventrículo esquerdo (FEVE) diminuída de 50% e sem patologia valvular significativa. Com um nível inicial de troponina I de 916 ng/L (intervalos de referência: 2,5-46 ng/L), chegou-se ao diagnóstico preliminar de lesão miocárdica.

Embora a FEVE diminuída, o nível elevado de troponina I e a dispneia pudessem ser atribuídos à taquicardia, uma angiografia coronária foi necessária para uma investigação etiológica devido ao histórico de tabagismo e à idade avançada do paciente. A angiografia coronariana, realizada pela artéria femoral direita, revelou oclusão total crônica da ACD distal com fluxo colateral retrógrado do sistema coronariano esquerdo. Não foram encontradas lesões obstrutivas no tronco da coronária esquerda (TCE), artéria descendente anterior (ADA) esquerda e artéria circunflexa (CX). Além disso, uma placa obstrutiva foi observada em uma AC bem desenvolvida, mas ainda de pequeno calibre (Vídeo 1-5). A primeira tentativa com o cateter Judkins direito 4 (JR4) resultou em visualização abaixo do ideal da ACD distal e seus ramos proximais devido a uma saída incomum do seio coronário direito e, portanto, antes de encerrar o estudo, fizemos mais uma tentativa de reconectar a ACD usando o cateter Amplatz esquerdo 1 (AL1) para garantir uma visualização mais clara da circulação distal e da extensão da lesão na AC. Após envolver o óstio da ACD com AL1, a circulação distal ficou claramente visível. Porém, o fluxo distal da AC estava comprometido, resultando em fluxo TIMI 1. Isso foi evidenciado pela coloração com contraste na área da lesão, conforme demonstrado na visão anterior de JR4 (Vídeo 6). Devido ao calibre pequeno da artéria e à ausência dos sintomas relatados, optou-se pela terapia medicamentosa. O paciente foi então transferido para a UCO em condição hemodinamicamente estável.

**Figura 2:**
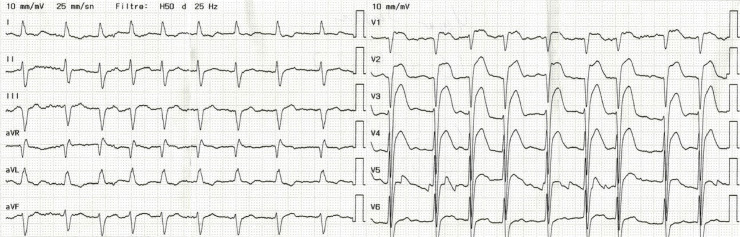
– O ECG após a primeira angiografia mostra características atribuíveis aos padrões de Brugada tipo 1 e tipo 2: Elevações do segmento ST nas derivações V1-4, assemelhando-se a um padrão em sela com uma onda T negativa como no Tipo 2, mas também com uma porção terminal do segmento ST gradualmente descendente, como no Tipo 1.

Após a transferência para a UCO, anormalidades no ECG, consistentes com características atribuíveis aos padrões de Brugada tipo 1 e tipo 2, com elevações de ST nas derivações V1-4, foram observadas no acompanhamento pós-procedimento de rotina (Figura 2). Embora o paciente ainda não relatasse angina, ele foi imediatamente encaminhado de volta ao laboratório de cateterismo para nova avaliação angiográfica em caso de piora do quadro ou desenvolvimento de nova oclusão coronariana.

A via de acesso foi alterada para artéria radial direita antes da intervenção devido aos desafios na manipulação do cateter causados pela tortuosidade da artéria femoral visualizada durante a captura de imagem anterior. Primeiramente, a angiografia coronária esquerda foi repetida e mostrou que o sistema coronário esquerdo estava inalterado e pérvio. No entanto, a repetição da imagem coronariana direita revelou que a oclusão subtotal anterior do ramo do cone havia progredido para oclusão total sem fluxo distal (Vídeo 7-8). Após a decisão de intervir, foram feitas múltiplas tentativas de reentrada no lúmen verdadeiro usando a técnica “buddy wire” e diversos fios-guia diferentes. No entanto, o fluxo não foi alcançado. Com base na relação risco-benefício e na ausência de angina ou arritmia maligna, não foram feitas novas tentativas e optou-se por continuar com a terapêutica medicamentosa. O paciente iniciou infusão de inibidor da glicoproteína IIb/IIIa (tirofiban) e, posteriormente, retomou a terapia antitrombótica tripla oral (ácido acetilsalicílico, varfarina e clopidogrel).

**Figura 3:**
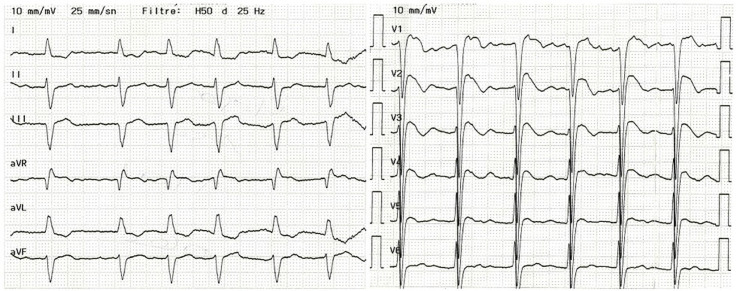
– No dia seguinte, o ECG mostra diminuição da elevação do segmento ST e padrão de Brugada mais pronunciado.

**Figura 4:**
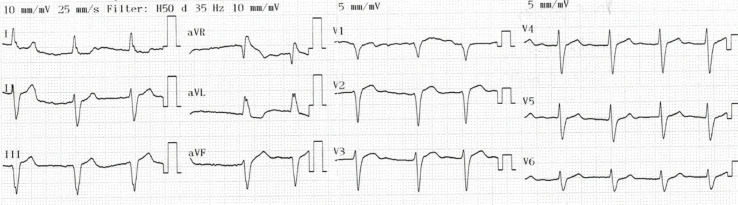
– ECG de alta sem sinal de padrão de Brugada.

As elevações do segmento ST diminuíram e o padrão de Brugada tornou-se mais proeminente no dia seguinte ao procedimento (Figura 3). O paciente não apresentou sintomas ou arritmias durante o restante da permanência na UCO ou posteriormente na enfermaria do hospital. Posteriormente, recebeu alta em condição estável, sem padrão de Brugada (Figura 4).

## Discussão

A AC, normalmente o ramo inicial da ACD, não somente supre sangue à VSVD, mas também desempenha um papel na circulação colateral no caso de oclusões na árvore coronária. Diversos mecanismos foram relatados para interromper o fluxo sanguíneo para a AC, incluindo prisão durante a colocação do stent em uma lesão proximal da ACD,^4^ vasoespasmo induzido por injeção de acetilcolina^3^ e intubação seletiva inadvertida seguida de injeção de corante de contraste na AC.

O caso apresentado aqui possui características únicas que o distinguem dos demais: 1) Este é o primeiro caso de oclusão de AC causada por dissecção de cateter, resultando em elevação ântero-septal do segmento ST com características dos padrões de ECG de Brugada tipo 1 e tipo 2. Em um estudo anterior sobre padrões de Brugada associados à isquemia, um padrão flutuante também foi relatado na maioria dos pacientes (5 de 6);^5^ 2) O paciente não relatou quaisquer sintomas anginosos ou eventos arrítmicos durante ou após a oclusão da artéria coronária (AC). Isto contrasta com o conhecimento convencional de que mesmo uma breve interrupção no fluxo sanguíneo da AC pode causar FV.^4,6^ Diversos fatores podem explicar esta observação: Primeiramente, o fluxo sanguíneo colateral do sistema coronário esquerdo devido à oclusão total crônica (OTC) da ACD distal poderia ser a causa disso. Outra possível explicação para a ocorrência de TV/FV poderia ser o pré-condicionamento isquêmico devido ao histórico de síndrome coronariana crônica (SCC) do paciente. Esta hipótese é apoiada por um estudo que encontrou maior incidência de TV/FV em pacientes sem SCC pré-existente;^7^ 3) A oclusão da AC foi provavelmente causada por uma dissecção iatrogênica com o cateter AL1, conhecido por ter maior risco de causar tais incidentes em comparação com outras causas mencionadas no parágrafo anterior; 4) Foi levantada a hipótese de que a isquemia do VD, sem envolvimento inferior-posterior do VE, improvável na presença de circulação coronária dominante direita, resultaria em elevação do segmento ST devido à não correspondência das forças elétricas dominantes do infarto do VD.^8^ Até onde sabemos, não existem casos relatados na literatura desse fenômeno ocorrendo na presença de oclusão do cone com OTC distal na circulação coronária codominante. A elevação ântero-septal do segmento ST observada no caso aqui descrito pode ser parcialmente explicada pela proximidade da área afetada (VSVD) à parede torácica e pela AC relativamente bem desenvolvida.

Nem todas as obstruções de infartos de AC ou VSVD causam padrões semelhantes aos de Brugada. Quando isso ocorre, as elevações de ST tendem a ser menos pronunciadas em comparação com aquelas observadas na verdadeira síndrome de Brugada.^5^ Em contraste, nem todos os padrões de Brugada resultantes da isquemia são causados por oclusão da artéria coronária, uma vez que também foram relatados em oclusões de outras artérias coronárias.^9^ Embora o mecanismo responsável pela elevação distinta do segmento ST observada em pacientes com Brugada ainda não seja totalmente compreendido, um estudo sistêmico de pacientes sintomáticos com síndrome de Brugada revelou uma correlação entre isquemia e elevação acentuada do segmento ST seguida de FV. Isto pode ser devido à isquemia e à reentrada da fase 2.^10^ Com base nesses achados, sugere-se que pacientes com formas congênitas ou possivelmente adquiridas da síndrome de Brugada podem apresentar maior risco de morte súbita cardíaca por isquemia.^11^

O caso descrito aqui pode ser considerado como um paciente com padrão de ECG de Brugada com os achados atuais, ou pode ser considerado como “fenocópia de Brugada”, um conceito usado para descrever formações que possuem os mesmos padrões de ECG da verdadeira síndrome congênita de Brugada, mas são causadas por outros fatores clínicos, como isquemia miocárdica ou anormalidades metabólicas. Entretanto, ainda não é amplamente aceito na comunidade científica. De acordo com o chamado “sistema de classificação morfológica da fenocópia de Brugada”, o paciente se enquadra na “fenocópia de Brugada Classe C de Tipo 1 ou 2”. Isso ocorre porque o paciente não possui antecedentes pessoais ou familiares que sugiram síndrome de Brugada. Assim, um desafio medicamentoso não foi realizado neste caso devido à baixa probabilidade pré-teste.^12^

## Conclusão

O caso aqui descrito destaca o fato de que a oclusão aguda da AC pode imitar a morfologia anterior do IAMCSST em um ECG, sendo tipicamente causada por oclusões no sistema coronariano esquerdo. Além disso, o padrão de Brugada, por vezes, pode ser observado em oclusões da AC. Por fim, é fundamental ter cautela ao usar cateteres AL, principalmente quando se trata de oclusões de ramos proximais. Mais pesquisas são necessárias para comparar pacientes com síndrome de Brugada verdadeira sobreposta com aqueles que apresentam padrão de Brugada genuíno induzido por isquemia.
