# Supplementary material for: Elevação Enganosa do Segmento ST Ântero-Septal e Padrão de Brugada Causado por Oclusão Isolada da Artéria do Cone
Source: Arq Bras Cardiol. 2024 Jul 1;121(6):e20230749. [Article in Portuguese] doi: 10.36660/abc.20230749 (PMC12080716; doi:10.36660/abc.20230749)
Supplement: Translation

# Translation: Deceptive Anteroseptal ST-segment Elevation and Brugada Pattern Caused by Isolated Conus Artery Occlusion

**DOI:** 10.36660/abc.20230749i

**Authors:** Hakan Gökalp Uzun, İrfan Özgen

**Affiliations:** 1 Turkey Izmir Tepecik Training and Research Hospital – Department of Cardiology, Konak, İzmir – Turkey

**Keywords:** Ventricular Outflow Obstruction, Right, ST Elevation Myocardial Infarction, Brugada Syndrome

## Abstract

The conus artery (CA) supplies the right ventricular outflow tract (RVOT). ST-segment elevation in leads V1-3, which can resemble Brugada electrocardiogram (EKG) patterns, has been reported due to occlusion of the CA. A 68-year-old male was admitted to the hospital with a diagnosis of non-ST-elevation myocardial infarction. A coronary angiogram revealed a dissection in the conus artery, most likely caused by the catheter. Due to the small caliber of the CA, medical therapy was chosen as the course of action. However, after the procedure, an EKG showed changes consistent with features of both type-1 and type-2 Brugada patterns, with ST-segment elevations in leads V1-4. Subsequent coronary imaging revealed that the CA had progressed to total occlusion. Despite multiple attempts to gain reentry into the true lumen, they were unsuccessful. Based on the risk-benefit ratio, the decision was made to continue with medical therapy. This is the first reported case of CA occlusion induced by catheter dissection, which manifested as anteroseptal ST-segment elevation. The patient did not report any anginal symptoms or arrhythmic events, which contrasts with conventional knowledge. Not all CA obstructions or RVOT infarcts cause Brugada-like patterns. When they do, ST elevations tend to be less than those in true Brugada syndrome.

## Introduction

The conus artery (CA), often the first branch of the right coronary artery (RCA) with a prevalence of 80.5%, supplies the outflow tract or conus arteriosus (infundibulum) of the right ventricle (RV), a region thought to be prone to generate arrhythmias.^1,2^

ST-segment elevation in leads V1-3, sometimes resembling Brugada EKG patterns, has been reported due to disruption of the blood flow to the CA via various mechanisms.^3^

## Case Report

A 68-year-old male ex-smoker with no history of chronic disease or drug use was admitted to the coronary care unit (CCU) with complaints of respiratory distress, palpitations, and leg swelling for 10 days.

Vital signs were normal except for an irregular and rapid heart rate, which was later confirmed on EKG to be consistent with atrial fibrillation with a rapid ventricular response. The admission EKG revealed atrial fibrillation, poor R-wave progression in the precordial leads, left ventricular overload, and left anterior fascicular block (Figure 1). Rales confined to the lung bases were audible on auscultation. The transthoracic echocardiogram, suboptimal due to underlying tachycardia, showed mild global hypokinesia with a depressed left ventricular ejection fraction (LVEF) of 50% and no significant valvular pathology. With an initial troponin I level of 916 ng/L (reference ranges: 2.5-46 ng/L), a preliminary diagnosis of myocardial injury was made.

**Figure 1:** – Admission EKG showed atrial fibrillation, poor R-wave progression in the precordial leads, left ventricular overload, and left anterior fascicular block.

Although the depressed LVEF, elevated troponin I level, and dyspnea could be attributed to tachycardia, a coronary angiogram was warranted for an etiologic workup due to the patient’s smoking history and advanced age. The coronary angiogram, performed via the right femoral artery, revealed chronic total occlusion of the distal right coronary artery with retrograde collateral flow from the left coronary system. No obstructive lesions were found in the left main coronary artery (LMCA), left anterior descending artery (LAD), and circumflex artery (CX). Additionally, an obstructive plaque was noted in a well-developed but still small-caliber CA (Video 1-5). Because the first attempt with the Judkins right 4 (JR4) catheter resulted in less-than-optimal visualization of the distal RCA and its proximal branches due to an unusual exit from the right coronary sinus before terminating the study, we made one more attempt to reengage the RCA using the Amplatz left 1 (AL1) catheter to ensure clear visualization of the distal circulation and the extent of the lesion in the CA. After engaging the RCA ostium with AL1, the distal circulation was clearly visible. However, the distal flow of the CA was compromised, resulting in a TIMI 1 flow. This was evidenced by contrast dye staining in the lesion area, as demonstrated in the earlier view by JR4 (Video 6). Due to the small caliber of the artery and the absence of the reported symptoms, the medical therapy was chosen. The patient was then transferred to the CCU in a hemodynamically stable condition.

After the transfer to the CCU, EKG abnormalities consistent with features attributable to both type-1 and type-2 Brugada patterns with ST elevations in leads V1-4 were noted at routine post-procedural follow-up (Figure 2). Although the patient was still angina-free, he was immediately sent back to the catheterization laboratory for repeat angiographic evaluation in case his condition worsened, or a new coronary occlusion developed.

**Figure 2:** – The EKG after the first angiogram shows features attributable to both type-1 and type-2 Brugada patterns: ST-segment elevations in leads V1-4 resembling a saddle-back pattern with a negative T wave as in Type 2, but also with a gradually descending terminal portion of the ST-segment as in Type 1.

The access route was changed to the right radial artery before the intervention due to challenges in catheter manipulation caused by the tortuosity of the femoral artery experienced during the previous imaging. Firstly, the left coronary angiogram was repeated, and it showed that the left coronary system was unchanged and patent. However, repeat right coronary imaging revealed that the previous subtotal occlusion of the conus branch had progressed to total occlusion with no distal flow (Video 7-8). After the decision was made to intervene, multiple attempts were made to gain re-entry to the true lumen using the buddy wire technique and several different guidewires. However, the flow was not achieved. Based on the risk-benefit ratio and the absence of angina or malignant arrhythmia, no further attempts were made, and it was decided to continue with medical therapy. The patient was started on a glycoprotein IIb/IIIa inhibitor (tirofiban) infusion and then resumed with an oral triple anti-thrombotic therapy (acetylsalicylic acid, warfarin, and clopidogrel).

The ST-segment elevations decreased, and the Brugada pattern became more prominent the day after the procedure (see Figure 3). The patient did not experience any symptoms or arrhythmias for the rest of their stay in the CCU or later in the hospital ward. He was subsequently discharged in a stable condition without a Brugada pattern (refer to Figure 4).

**Figure 3:** – The following day EKG which shows decreased ST-segment elevations and a more pronounced Brugada pattern.

**Figure 4:** – Discharge EKG without sign of Brugada pattern.

## Discussion

The CA, typically the initial branch of the RCA, not only supplies blood to the RVOT but also plays a role in collateral circulation in the event of occlusions in the coronary tree. Several mechanisms have been reported to disrupt blood flow to the CA, including jailing during stent placement in a proximal RCA lesion,^4^ vasospasm induced by acetylcholine injection,^3^ and inadvertent selective intubation followed by injection of contrast dye into the CA.

Our case has unique features that distinguish it from others: 1) This is the first case of CA occlusion caused by catheter dissection, resulting in anteroseptal ST-segment elevation with features of both type-1 and type-2 Brugada EKG patterns. In a previous study on Brugada patterns associated with ischemia, a fluctuating pattern was also reported in most patients (5 out of 6);^5^ 2) The patient did not report any anginal symptoms or arrhythmic events during or after the occlusion of the coronary artery (CA). This contrasts with conventional knowledge that even a brief interruption to CA blood flow could cause VF.^4,6^ Several factors could explain this observation: Firstly, the collateral blood flow from the left coronary system due to chronic total occlusion (CTO) of the distal RCA would have caused this. Another possible explanation for the occurrence of VT/VF could be ischemic preconditioning due to the patient’s history of chronic coronary syndrome (CCS). This hypothesis is supported by a study that found a higher incidence of VT/VF in patients without pre-existing CCS;^7^ 3) The occlusion of the CA was likely caused by an iatrogenic dissection with the AL1 catheter, which is known to have a higher risk of causing such incidents compared to other causes mentioned in the previous paragraph; 4) It has been hypothesized that RV ischemia without inferior-posterior LV involvement, which is unlikely in the presence of right-dominant coronary circulation, would result in anteroseptal ST-segment elevation due to non-matching of the dominant electrical forces from the RV infarction.^8^ To the best of our knowledge, there are no reported cases in the literature of this phenomenon occurring in the presence of conus occlusion with a distal CTO in the codominant coronary circulation. The anteroseptal ST-segment elevation in our case may be partially explained by the proximity of the affected area (RVOT) to the chest wall and the relatively well-developed culprit CA.

Not all obstructions of CA or RVOT infarcts cause Brugada-like patterns. When they do, ST elevations tend to be less pronounced than those seen in true Brugada syndrome.^5^ In contrast, not all Brugada patterns due to ischemia are caused by coronary artery occlusion; they have also been reported in occlusions of other coronary arteries.^9^ Although the mechanism responsible for the distinctive ST-segment elevation observed in Brugada patients is not yet fully understood to date, a systemic study of symptomatic patients with Brugada syndrome has revealed a correlation between ischemia and marked ST-segment elevation followed by VF. This may be due to ischemia and phase 2 reentry.^10^ Based on these findings, it is suggested that patients with congenital or possibly acquired forms of Brugada syndrome may be at a higher risk of sudden cardiac death caused by ischemia.^11^

Our case can be considered as a patient with Brugada EKG pattern with the current findings, or it can be considered as ‘Brugada phenocopy,’ a concept used to describe formations that have the same EKG patterns as true congenital Brugada syndrome but are caused by various other clinical factors such as myocardial ischemia or metabolic abnormalities. However, it is not yet widely accepted in the scientific community. According to the so-called ‘Brugada phenocopy morphologic classification system,’ the patient falls under the ‘Type-1 or 2, Class C Brugada phenocopy’. This is because the patient has no personal or family history that suggests Brugada syndrome. Accordingly, a drug challenge was not performed in our case due to the low pretest probability.^12^

## Conclusion

Our case highlights the fact that acute occlusion of the CA can imitate the anterior STEMI morphology on an EKG,

which is typically caused by occlusions in the left coronary system. Additionally, the Brugada pattern may sometimes be observed in conus artery occlusions. Finally, it is crucial to exercise caution when using AL catheters, particularly when dealing with proximal branch occlusions. Further research is necessary to compare patients with overlapping true Brugada syndrome with those who have a genuine ischemia-induced Brugada pattern.
